# Integrative Bioinformatics Approaches to Screen Potential Prognostic Immune-Related Genes and Drugs in the Cervical Cancer Microenvironment

**DOI:** 10.3389/fgene.2020.00727

**Published:** 2020-07-07

**Authors:** Zitong Zhao, Jigang Li, He Li, Na-Yi Yuan Wu, Peilin Ou-Yang, Shan Liu, Jingting Cai, Jing Wang

**Affiliations:** ^1^Hunan Cancer Hospital/The Affiliated Cancer Hospital of Xiangya School of Medicine, Central South University, Changsha, China; ^2^Department of Medicine, University of South China, Hengyang, China

**Keywords:** cervical cancer, tumor microenvironment, TCGA, GEO, multifactor, drug

## Abstract

In developing countries, cervical cancer is still the major cause of cancer-related death among women. To better understand the correlation between tumor microenvironment (TME) and prognosis of cervical cancer, we screened 1367 differentially expressed genes (DEGs) of cervical cancer samples in The Cancer Genome Atlas (TCGA) database using Estimation of STromal and Immune cells in MAlignant Tumor tissues using Expression data (ESTIMATE) algorithm-derived immune scores. Then, we extracted 401 tumor immune microenvironment (TIME)-related DEGs that related to patients’ survival outcomes. Protein-protein interaction (PPI) network and functional enrichment analysis revealed that the prognostic genes mainly participated in myeloid leukocyte activation, adaptive immune response regulation, and receptor signaling pathways. A total of 79 key prognostic DEGs were obtained through PPI network. A TF-lncRNA-miRNA-mRNA regulatory network was constructed to explore the potential regulatory mechanism. 4 genes (*CCR7*, *PD-1*, *ZAP70*, and *CD28*) were validated in another independent cohort of cervical cancer from the Gene Expression Omnibus (GEO) database. Finally, potential drugs for key prognostics DEGs were predicted using DrugBank. In conclusion, we obtained a list of potential prognostic TIME-related genes and potential predicted drugs by integrative bioinformatics approaches. A comprehensive understanding of prognostic genes within the TIME may provide new strategies for cervical cancer treatment.

## Introduction

In developing countries, cervical cancer is still the major cause of cancer-related death among women (Arbyn et al., 2020). Nearly all cervical cancers are associated with human papillomavirus (HPV) infection (Berman and Schiller, 2017). Although significant progress has been achieved in screening and prevention, the 5-year overall survival (OS) rate for cervical cancer remains around 60% (McLachlan et al., 2017). Radiotherapy and chemotherapy are standard therapies for advanced-stage patients (Wui-Jin et al., 2019), but with limited success. Recently, remarkable progress in cervical cancer immunotherapy has been made, but positive responses only occur in a small fraction of patients. Such responses are usually dependent on dynamic interactions between tumor cells and other factors within the tumor microenvironment (TME).

Tumor microenvironment contains tumor cells and the surrounding blood vessels, signaling molecules, immune cells, and fibroblasts (Joyce and Fearon, 2015; Spill et al., 2016), etc. The TME can critically influence gene expression in cancer tissues, and is gradually recognized as a key contributor to cancer progression and drug resistance (Piersma, 2011; Pasini et al., 2014; Kim et al., 2016; Petitprez et al., 2018; Li et al., 2019; Looi et al., 2019). Cancer cells can create a full range of immunosuppression in the TME to counter the body’s anti-tumor immunity and achieve immune escape (Teng et al., 2015). Several studies have suggested that tumor-associated macrophages (TAMs), matrix metalloproteinase, transforming growth factor-beta, and interleukin (IL)-2 play key roles in cervical cancer progression and are associated with cancer cell invasion and dissemination ability (Valle-Mendiola et al., 2016; Zhu et al., 2016; Ng et al., 2019; Wang et al., 2019a). A deep understanding of the correlation between TME and prognosis, and exploring new strategies for the treatment are urgently needed for precise therapy improvement of cervical cancer.

**FIGURE 1:**
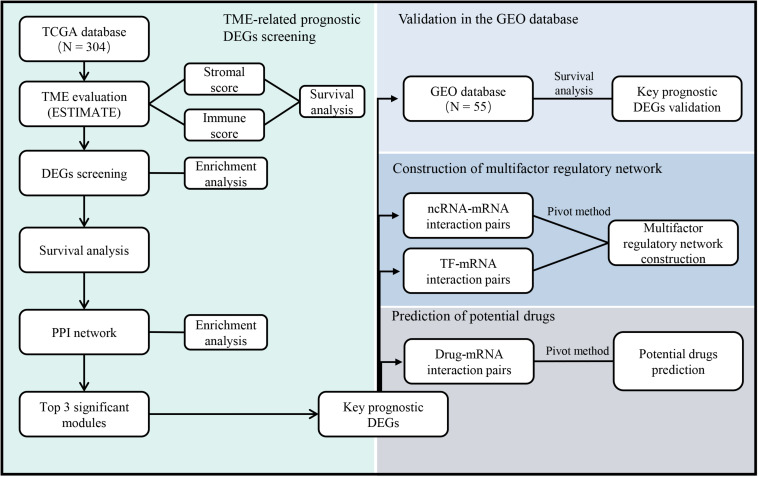
The workflow of the current study. TCGA = The Cancer Genome Atlas, GEO = Gene Expression Omnibus, TME = Tumor microenvironment, DEGs = Differentially expressed genes, PPI = Protein-protein interaction, ncRNA = non-coding RNA, TF = Transcription factor.

With the rapid development of public databases and second-generation sequencing technologies, comprehensive analysis for TME-related prognostic genes has become possible. The Estimation of STromal and Immune cells in MAlignant Tumor tissues using Expression data (ESTIMATE) algorithm (Yoshihara et al., 2013) was developed to predict infiltrating immune and stromal cells within tumor tissues using gene expression data in The Cancer Genome Atlas (TCGA) database. Subsequent studies have involved the ESTIMATE algorithm to glioblastomas (Jia et al., 2018), renal cell carcinomas (Xu et al., 2019), and colon cancers (Alonso et al., 2017). However, the utility of the ESTIMATE algorithm in cervical cancer has not been previously investigated. In this study, we screened the expression and interaction of TME-related differentially expressed genes (DEGs) in cervical cancer, predicted their regulatory network, and evaluated the potential therapeutic drugs based on several large public databases (Figure 1). The results might provide useful clues for prospective treatment strategies of cervical cancer.

## Materials and Methods

### Data

Gene expression profiles and related clinical materials for cervical cancer were downloaded from the TCGA data portal (Tomczak et al., 2015). The inclusion criteria were (Arbyn et al., 2020) pathologically confirmed cervical cancer, (Berman and Schiller, 2017) complete RNA expression data from the patients, and (McLachlan et al., 2017) complete ESTIMATE score, immune score, and stromal score (Yoshihara et al., 2013).

For further verification, gene expression profiles and clinical materials of another cohort of cervical cancer patients were downloaded from the Gene Expression Omnibus (GEO) database (GSE52903) (Medina-Martinez et al., 2014). In addition, we also used an online web server (OScc) to verify the prognostic value of targeted genes (Wang et al., 2019b).

### Identification of DEGs and Functional Enrichment Analysis

Data analysis was conducted using package limma in R language (version 3.4.0) (Ritchie et al., 2015). A fold change (FC) > 2 and adjusted *p*-value < 0.05 were set up to screen DEGs. Heat maps were generated by pheatmap package in R (Kolde and Kolde, 2015).

Through the Search Tool for Retrieval of Interacting Genes/Proteins (STRING) database (version 11.0), functional enrichment analysis was conducted to identify gene ontology (GO) annotation (Szklarczyk et al., 2019) and Kyoto Encyclopedia of Genes and Genomes (KEGG) pathways. *P* < 0.05 was considered to be statistically significant.

### Survival Analysis

Using the survival and survminer package in R, Kaplan-Meier plots and log-rank tests were performed to elucidate the relationship between 5-year overall survival (OS) rates and DEGs expression levels. Univariate Cox regression was used to assess the effect of clinical parameters and mRNA expression on the survival of cervical cancer patients. *P* < 0.05 was considered to be statistically significant.

### Protein-Protein Interaction (PPI) Network Building and Gene Set Enrichment Analysis (GSEA)

The PPI network was extracted from the STRING database and visualized by Cytoscape software (version 3.4.0) (Shannon et al., 2003). To identify densely connected regions, Molecular COmplex Detection (MCODE) in Cytoscape was then involved to extract topology-based clusters.

Using the STRING database and GSEA method, we further retrieved a functional profile of the gene set derived from the PPI network (Mootha et al., 2003; Subramanian et al., 2005). *P* < 0.05 was considered to be statistically significant.

### Extraction of microRNA (miRNA), Long Non-coding RNA (lncRNA), Transcription Factor (TF), and Drug Interactions

We obtained the miRNA – mRNA and lncRNA-mRNA interactions from the RNA Interactome (RNAInter) database (version RNAInter in 2020) (Lin et al., 2019), TF-mRNA interactions from the Transcriptional Regulatory Relationships Unraveled by Sentence-based Text mining (TRRUST) database (version 2.0) (Han et al., 2017), and drug-mRNA interactions from the DrugBank database (version 5.1.1) (Law et al., 2014). RNAInter, TRRUST V2 and DrugBank include the curated confirmed interactions from the literatures.

To construct a muti-factor regulator network, we extracted miRNAs, lncRNAs, TFs, and drugs that had interactions with obtained genes.

### Pivot Method

We further screened pivot nodes from obtained interaction pairs using the *phyper()* function in R. The pivot node refers to at least two interacting pairs between the node and a gene, and the significance analysis *p*-value of the interaction between the node and the gene set should be <0.05 by the hypergeometric test (Wu et al., 2015). The obtained pivot miRNAs-mRNAs, lncRNAs-mRNAs, and TFs-mRNAs interactions were visualized by Cytoscape. Pivot drugs-mRNA interactions pairs were also analyzed.

## Results

### Immune Scores Are Significantly Associated With HPV Infection, Histological Type, and Patients’ Survival

**FIGURE 2:**
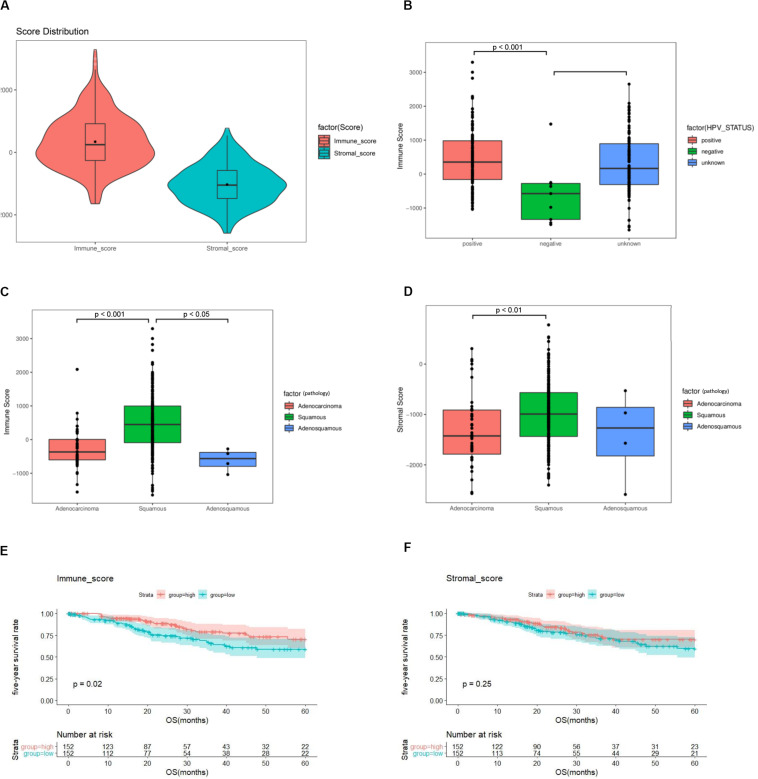
The immune score and stromal score are associated with clinicopathologic characteristics and overall survival of cervical cancer patients. **(A)** Distribution of immune scores and stromal scores among 304 cervical cancer samples in TCGA. **(B)** Distribution of immune scores among HPV-negative and HPV-positive cases. **(C)** Distribution of immune scores among cervical cancer subtypes. **(D)** Distribution of stromal scores among cervical cancer subtypes. **(E)** A higher immune score is associated with better overall survival (*p* = 0.02). **(F)** Stromal score is not associated with overall survival (*p* = 0.25). TCGA = The Cancer Genome Atlas, GEO = Gene Expression Omnibus, HPV = Human papillomavirus.

Among 304 cases in TCGA, 253 (83.2%) were squamous carcinomas, 47 (15.5%) adenocarcinomas, and 4 (1.3%) adenosquamous carcinomas (Supplementary Table 1). Based on the ESTIMATE algorithm, the median of stromal scores was -1047.855 (-2586.99 to 778.01), and the median of immune scores was -246.78 (-1645.63 to 3295.3) (Figure 2A). HPV-positive cases had higher immune scores than HPV-negative cases (*p* < 0.001) (Figure 2B). Cases of squamous carcinoma had significantly higher immune scores and stromal scores than cases of adenocarcinoma (*p* < 0.01) (Figures 2C,D).

To assess the potential relationship of stromal and immune scores with patients’ outcome, a total of 304 cervical cancer cases were categorized into high-score and low-score groups by the median expression value. The results revealed that patients with high immune scores had a better survival outcome than those with low scores (*p* = 0.02) (Figure 2E). There was no difference in survival outcomes between the two stromal-score groups (*p* = 0.25) (Figure 2F).

### DEG Screening and Functional Analysis Between Low- and High-Immune Score Groups

**FIGURE 3:**
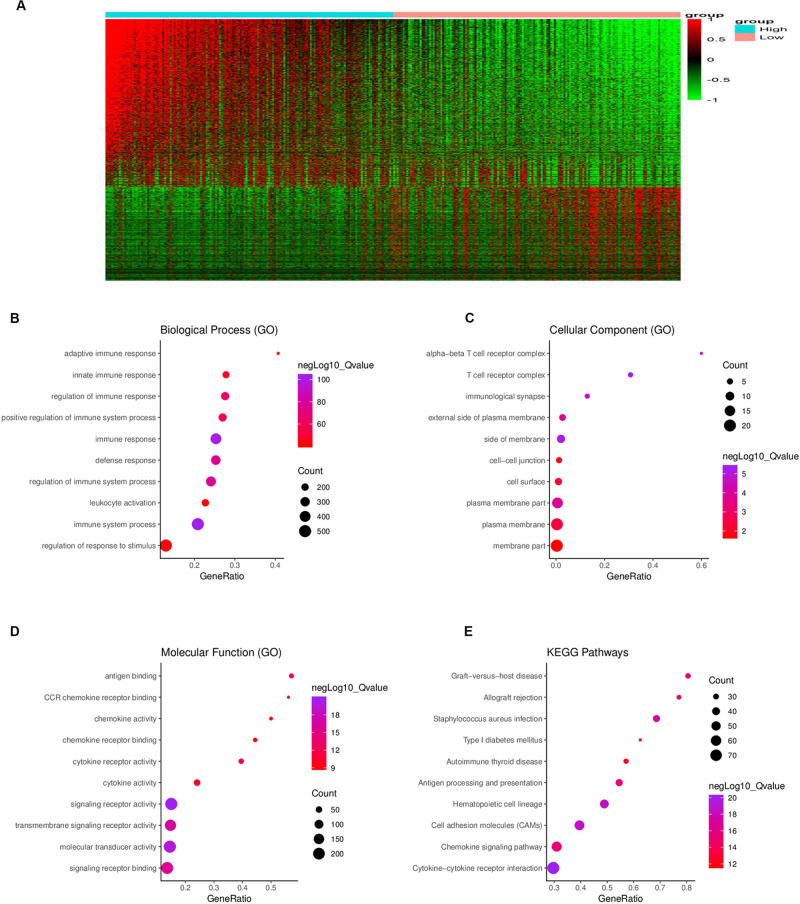
Comparison of gene expression profiles between the high- and low-immune score groups. **(A)** In the heat maps, genes with higher expression are shown in red, and genes with lower expression are shown in green; genes expressed at the same level are in black. A total of 879 genes were upregulated and 488 genes downregulated in the high-score group as compared to the low-score group. Biological process **(B)**, cellular component **(C)**, and molecular function **(D)** in gene ontology (GO) analysis for 1367 DEGs. **(E)** Kyoto Encyclopedia of Genes and Genomes (KEGG) analysis for 1367 DEGs. DEG = differentially expressed gene.

To determine the relationship between global gene expression profiles and immune scores, 1367 DEGs between the two immune-score groups were identified, including 488 downregulated genes and 879 upregulated genes (Figure 3A).

To further understand the potential biological function of the DEGs, GO annotation and KEGG pathway were analyzed. GO analysis showed that the DEGs were mainly enriched in ingredients such as immunological synapse and T cell receptor complex, and mainly enriched in processes such as immune system process and regulation of immune system process, signaling receptor binding, and leukocyte activation (Figures 3B–D). KEGG pathway enrichment analysis demonstrated that the DEGs were mainly associated with antigen processing and presentation, cytokine-cytokine receptor interaction, chemokine signaling pathway and cell adhesion molecules, etc. (Figure 3E).

**FIGURE 4:**
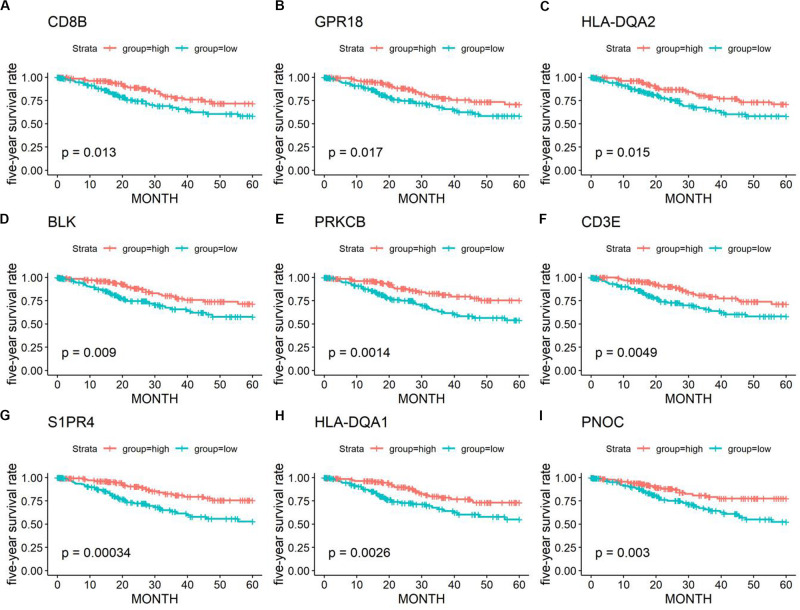
Kaplan–Meier survival curves and log-rank tests showing the correlation of partial immune-related genes **(A–I)** with the 5-year survival rates. High gene expression (red line) was correlated with better outcomes in cervical cancer patients.

Kaplan–Meier plots were further performed for 1367 DEGs. A total of 401 DEGs were significantly related to the 5-year OS (Figure 4) (Supplementary Table 2).

### PPI Networks Construction and Functional Enrichment Analysis

**FIGURE 5:**
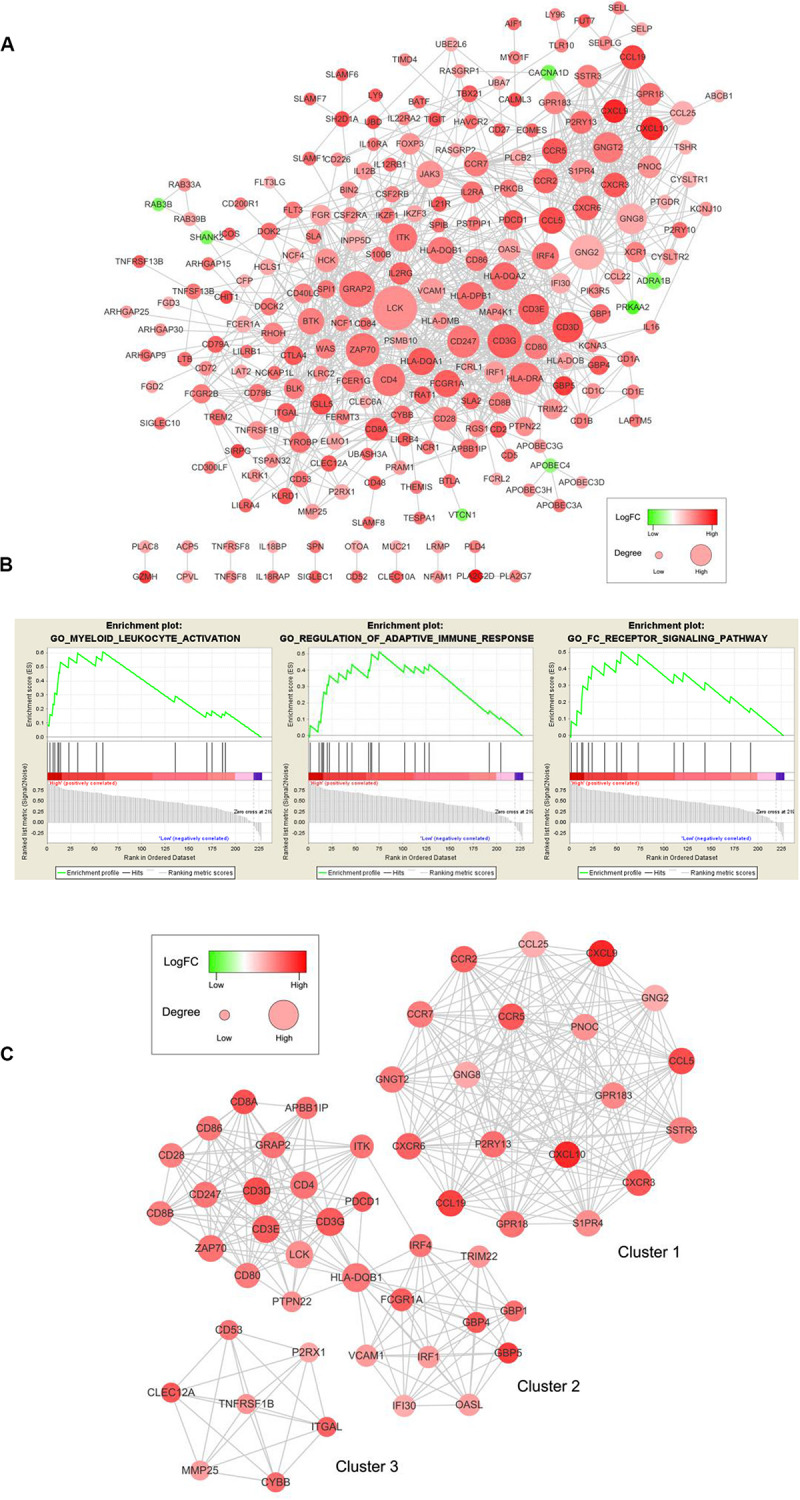
PPI network among prognostic genes and functional enrichment analysis. **(A)** The PPI network was made up of 15 modules and included 228 nodes and 1041 edges. The color of a node in the PPI network reflects the log (FC) value of the Z score of gene expression, and the size of the node indicates the number of proteins interacting with the designated protein. **(B)** Enrichment profiles generated with GSEA for the gene set in the PPI network. **(C)** Top three modules in the PPI network. PPI = protein-protein interaction, FC = Fold change, GSEA = gene set enrichment analysis.

To examine the interplay among the prognostic DEGs, we built a PPI network, which was made up of 15 modules and comprised 228 nodes and 1041 edges (Figure 5A). GSEA was used to clarify the main biological functions of 228 node genes. The results showed that they were mainly associated with myeloid leukocyte activation, adaptive immune response regulation, and receptor signaling pathways (Figure 5B).

We selected the top three significant modules for further analysis and named these modules cluster 1, cluster 2, and cluster 3 (Figure 5C). Cluster 1 had 171 edges and 19 nodes in the network. In cluster 2, *HLA-DQB1*, *CD3G*, *CD3D*, *CD4*, *CD3E*, *LCK*, and *ZAP70*, which are critical to the immune response, had higher degree values. In cluster 3, *TNFRSF1B*, which is crucial to immune and inflammatory procession (Croft, 2009), occupied the module center.

After selecting from the three modules and PPI networks with ≥10 node degrees, we obtained 79 key prognostic DEGs (Supplementary Table 3).

### Validation of Key Prognostic DEGs in the GEO Database

**FIGURE 6:**
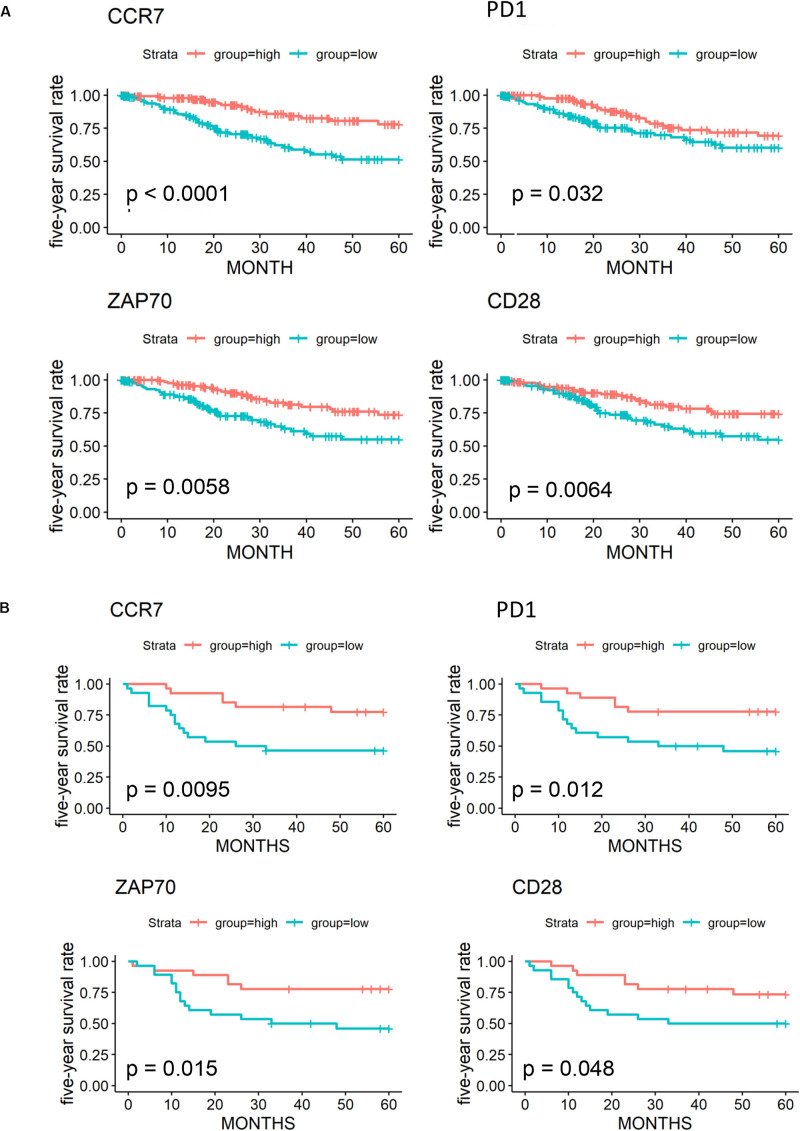
Validation of 79 key prognostic DEGs in an independent GEO cohort. **(A)** Kaplan-Meier plots and log-rank tests in the TCGA cohort were performed for 4 validated prognostic DEGs based on high (red line) and low (blue line) gene expression. **(B)** Kaplan–Meier plots and log-rank tests in the GEO cohort were performed for 4 validated prognostic DEGs.

We further validated 79 key DEGs in another cohort of 55 cervical cancer patients from the GEO database. Finally, high expressions of four genes (*CCR7*, programmed cell death-1 [*PD-1*], *ZAP70*, and *CD28*) was found to be associated with better 5-year OS in both GEO (Figures 6A,B) and OScc (Supplementary Figure 1). In univariate analysis, high expression of *CCR7, PD-1*, and *ZAP70* were related to better survival outcome in both TCGA and GEO (Supplementary Table 4).

### Construction of a Multifactor Regulatory Network Based on Key Prognostic DEGs

**FIGURE 7:**
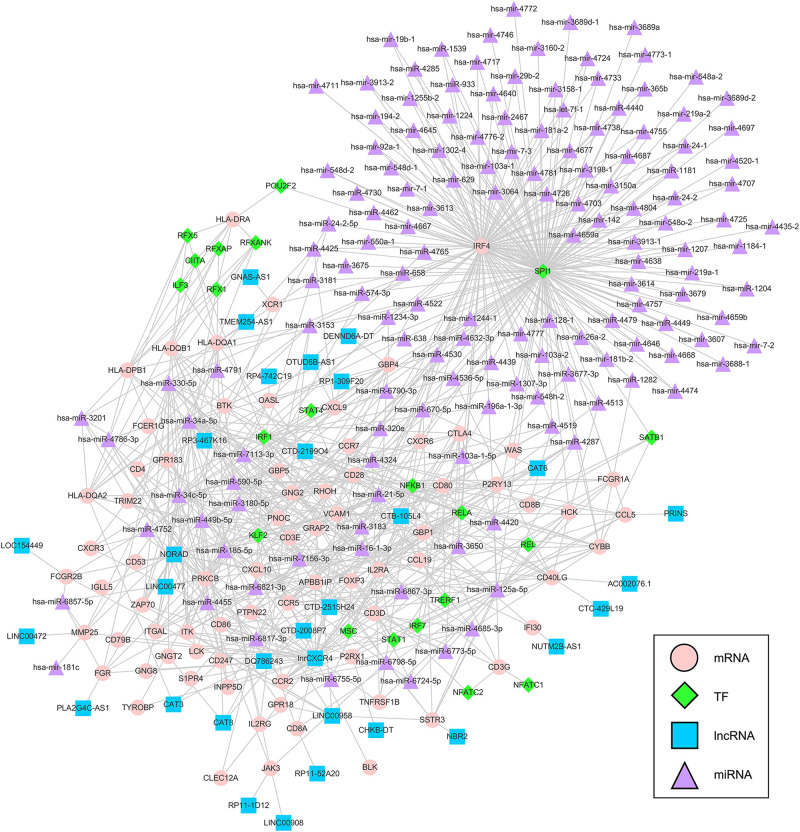
Multifactor regulatory network of key prognostic DEGs. The network was composed of 148 pivot miRNAs, 31 pivot lncRNAs, 21 pivot TFs, and 75 pivot mRNAs. TF = transcription factor, lncRNA = long non-coding RNAs, miRNA = microRNA.

We extracted interaction pairs of miRNAs, lncRNAs, and TFs with 79 key DEGs and constructed a multifactor regulatory network. The network contained 2295 nodes and 7678 edges (2058 miRNA nodes, 79 lncRNA nodes, 192 TF nodes, and 76 mRNA nodes). To acquire nodes with greater influence on the network, the network was pruned using the pivot method and visualized with Cytoscape. The final network including 31 pivot lncRNAs, 148 pivot miRNAs, 21 pivot TFs, and 75 pivot mRNAs, was identified (Figure 7).

### Identification of Potential Predictive Drugs

From DrugBank, we obtained 25020 drug-mRNA interaction pairs. A total of 79 key DEGs were then inputted into the database to predict the potential drugs of the genes, and 149 drug-mRNA interactions were extracted. Pivot method was used to simplify the obtained drugs and a total of 39 pivot drugs were predicted (Table 1). For example, Bevacizumab and cetuximab has been reported to target FCGR1A and FCGR2B (Imming et al., 2006; Bogdanovich et al., 2016), and these 2 drugs (Moore et al., 2012; Zighelboim et al., 2013; Tewari et al., 2014; Penson et al., 2015) have already been approved for clinical treatment of cervical cancer.

**TABLE 1:** Thirty-nine predicted potential drugs and their targeted DEGs in cervical cancer.

DrugBank ID	Drug	*p-*value	Gene
DB00075	Muromonab	< 0.001	*CD247, CD3D, CD3E, CD3G, FCGR1A, FCGR2B*
DB00098	Antithymocyte immunoglobulin	< 0.001	*CD4, CD86, FCGR2B, ITGAL*
DB06681	Belatacept	< 0.001	*CD80, CD86*
DB01281	Abatacept	< 0.001	*CD80, CD86*
DB00095	Efalizumab	0.001	*FCGR1A, FCGR2B, ITGAL*
DB00004	Denileukin diftitox	0.001	*IL2RA, IL2RG*
DB12698	Ibalizumab	0.001	*CCR5, CD4*
DB00111	Daclizumab	0.001	*FCGR1A, FCGR2B, IL2RA*
DB00074	Basiliximab	0.002	*FCGR1A, FCGR2B, IL2RA*
DB02010	Staurosporine	0.002	*ITK, LCK, ZAP70*
DB00005	Etanercept	0.003	*FCGR1A, FCGR2B, TNFRSF1B*
DB06607	Catumaxomab	0.005	*CD3E, FCGR1A*
DB00041	Aldesleukin	0.010	*IL2RA, IL2RG*
DB11767	Sarilumab	0.015	*FCGR1A, FCGR2B*
DB00112	Bevacizumab	0.019	*FCGR1A, FCGR2B*
DB00081	Tositumomab	0.019	*FCGR1A, FCGR2B*
DB00110	Palivizumab	0.019	*FCGR1A, FCGR2B*
DB00028	Immune Globulin Human	0.019	*FCGR1A, FCGR2B*
DB00087	Alemtuzumab	0.019	*FCGR1A, FCGR2B*
DB00092	Alefacept	0.019	*FCGR1A, FCGR2B*
DB00002	Cetuximab	0.022	*FCGR1A, FCGR2B*
DB00073	Rituximab	0.022	*FCGR1A, FCGR2B*
DB00078	Ibritumomab tiuxetan	0.022	*FCGR1A, FCGR2B*
DB00108	Natalizumab	0.022	*FCGR1A, FCGR2B*
DB00056	Gemtuzumab ozogamicin	0.022	*FCGR1A, FCGR2B*
DB00051	Adalimumab	0.022	*FCGR1A, FCGR2B*
DB00072	Trastuzumab	0.030	*FCGR1A, FCGR2B*
DB00054	Abciximab	0.030	*FCGR1A, FCGR2B*
DB01254	Dasatinib	0.022	*BTK, FGR, LCK*
DB00071	Insulin Pork	0.034	*HLA-DQA2*
DB00707	Porfimer sodium	0.039	*HLA-DQB1*
DB11714	Durvalumab	0.039	*CD80*
DB11626	Tasonermin	0.039	*TNFRSF1B*
DB05943	Resatorvid	0.039	*IL2RG*
DB04835	Maraviroc	0.039	*CCR5*
DB09052	Blinatumomab	0.039	*CD3D*
DB00895	Benzylpenicilloyl Polylysine	0.039	*FCER1G*
DB05501	AMD-070	0.039	*CCR5*
DB01809	Ter-Butyl-3-P-Tolyl-1h-Pyrazolo[3,4-D] Pyrimidin-4-Ylamine	0.039	*HCK*

## Discussion

Cervical cancer treatment has suffered rapid progress in the past decade. It moves away from drugs that attack tumors broadly toward precise immunotherapy that regulates immune responses against tumors. Identifying effective biomarkers related to tumor immune microenvironment (TIME) and prognosis are urgently needed for better patient management.

By ESTIMATE algorithm, we first found that high immune scores were related to better OS, which is consistent with the results of previous studies showing that immune cells infiltrating the tumor tissue may inhibit cancer cells (Cho et al., 2014; Gorter et al., 2015). The study also found HPV-positive cases had higher immune scores than HPV-negative cases, which might be associated with HPV-related microenvironment components regulation, such as increase of regulatory immune responses and decrease of effector immune responses (Zhou et al., 2019). A total of 1367 DEGs between the low- and high-immune score groups were identified, and 401 DEGs among them were related to survival outcomes of cervical cancer patients. These genes affect the outcomes of patients mainly by regulating TIME-related biological functions, including immune response regulation, leukocyte activation, chemokine activities, and integrin binding. These processes may shape tumor development and anti-cancer immunity, thus improving prognosis (Jochems and Schlom, 2011; Engblom et al., 2016; Böttcher et al., 2018).

A PPI network for 401 prognostic DEGs was constructed to reveal the interplay between DEGs, and 228 node genes were confirmed. The top modules that we selected from the PPI network have been reported to influence angiogenesis, proliferation, invasiveness, and therapeutic efficacy in cervical cancer (Yang et al., 2012; Zhang et al., 2015, 2018; Zhao et al., 2015; Che et al., 2016). The GSEA results showed that 228 node genes were highly associated with myeloid leukocyte activation, adaptive immune response regulation, and receptor signaling pathways. Silveira et al. reported that proliferation and accumulation of myeloid-derived suppressor cells might worsen cervical cancer progression and strong infiltration of CD14-positive myeloid cells might prolong survival in cervical cancer patients (Garcia et al., 2004; de Vos van Steenwijk et al., 2013).

By cross-validation with an independent GEO cohort, we identified four prognostic immune-related genes (*CCR7*, *CD28*, *PD-1*, and *ZAP70*). In previous studies, PD-1 expression was only found on the surface of immune cells, while programmed death receptor ligand-1 (PD-L1) was on cervical cancer cells. Their interaction played critical a role in tumor immune escape (Antoni, 2012). Monoclonal antibodies targeting PD-1/programmed death ligand, such as pembrolizumab, have already been widely assessed in clinical trials and are currently approved for the treatment of advanced cervical cancer (Borcoman and Le Tourneau, 2017; Chen et al., 2017; Chung et al., 2019; Dyer et al., 2019). Interestingly, in our results, higher expression of PD-1 was associated with better clinical outcomes. However, recent studies revealed a high intrinsic expression of PD-1 in most tumor cell lines (Yao et al., 2018). Combined with our functional enrichment analysis results of DEGs (myeloid leukocyte activation, adaptive immune response regulation, and receptor signaling pathways), we speculated that PD-1 expressed on tumor cells might have different functions, such as immune activation, other than that on immune cells. CCR7 has been reported to influence the lymph node metastasis of cervical cancer, prostate cancer cell migration, and mammary cancer cell stemness (Boyle et al., 2017; Dai et al., 2017; Maolake et al., 2018). Tyrosine kinase ZAP70 has been identified to play a key role in T cell activation and the immune response (Fu et al., 2016; Alsadeq et al., 2017; Laufer et al., 2018).

To explore the molecular mechanisms underlying the differential expression of these genes, we further constructed a TF-lncRNA-miRNA-mRNA regulatory network. We identified 148 pivot miRNAs, 31 pivot lncRNAs, 21 pivot TFs, and 75 pivot mRNAs. In addition, a total of 39 potential drugs for key prognostic DEGs were predicted. Bevacizumab was the first molecular antibody to show survival benefit in advanced cervical cancer, and it improved progression-free survival more than 3.7 months (Tewari et al., 2017). Cetuximab, an anti-epidermal growth factor receptor monoclonal antibody, is a standard option for the treatment of advanced cervical cancer (Meira et al., 2009). Fourteen drugs were identified, including catumaxomab, aldesleukin, trastuzumab, and ibritumomab tiuxetan, all of which have been confirmed for various cancers, including malignant ascites (Kietpeerakool et al., 2019), renal cell carcinoma (Fishman et al., 2019), gastric cancer (Kimura et al., 2018), and lymphoma (Lansigan et al., 2019), respectively. Among drug-interactions obtained, Staurosporine has been reported to target ZAP70 (Overington et al., 2006), but their interaction in cancer research is still blank.

One limitation of our study is that our predictions were based on analyses of online databases, so further experimental validation is needed. In future research, we will further explore the potential functions and signal pathways of the 79 DEGs (especially *CCR7*, *CD28*, *PD-1*, and *ZAP70*) within cervical cancer TIME. A deeper understanding of the complex molecular mechanism of TIME in cervical cancer may help explain the individual difference in immunotherapy efficiency and help explore new treatment strategies.

## Conclusion

We identified 79 prognostic TIME-related genes in cervical cancer and validated 4 genes (*CCR7*, *CD28*, *PD-1*, and *ZAP70*). Additionally, a total of 39 potential predicted drugs targeting key prognostic genes were obtained, and they might provide new clues for future treatment management. Further investigation of these genes and related regulatory network might put novel insights into the cervical cancer immunotherapy and prognosis improvement in a comprehensive manner.

## Data Availability Statement

Publicly available datasets were analyzed in this study, these can be found in The Cancer Genome Atlas (https://portal.gdc.cancer.gov/); the NCBI Gene Expression Omnibus (GSE52903).

## Author Contributions

ZZ, N-YY, JC, and JW performed the data analysis work and aided in writing the manuscript. ZZ and JW designed the study and edited the manuscript. JL, HL, PO-Y, and SL assisted in writing the manuscript. All authors read and approved the final manuscript.

## Conflict of Interest

The authors declare that the research was conducted in the absence of any commercial or financial relationships that could be construed as a potential conflict of interest.

## Abbreviations

DEG: differentially expressed gene
ESTIMATE: Estimation of STromal and Immune cells in MAlignant Tumor tissues using Expression data
GEO: Gene Expression Omnibus
HPV: human papillomavirus
lncRNA: long non-coding RNAs
miRNA: microRNA
OS: overall survival
PPI: protein-protein interaction
TCGA: The Cancer Genome Atlas
TF: transcription factor
TIME: tumor immune microenvironment
TME: tumor microenvironment.
