# Supplementary material for: Integrative Bioinformatics Approaches to Screen Potential Prognostic Immune-Related Genes and Drugs in the Cervical Cancer Microenvironment
Source: Front Genet. 2020 Jul 7;11:727. doi: 10.3389/fgene.2020.00727 (PMC7359727; doi:10.3389/fgene.2020.00727)
Supplement: Supplementary file 1 [file Data_Sheet_1.docx]

*
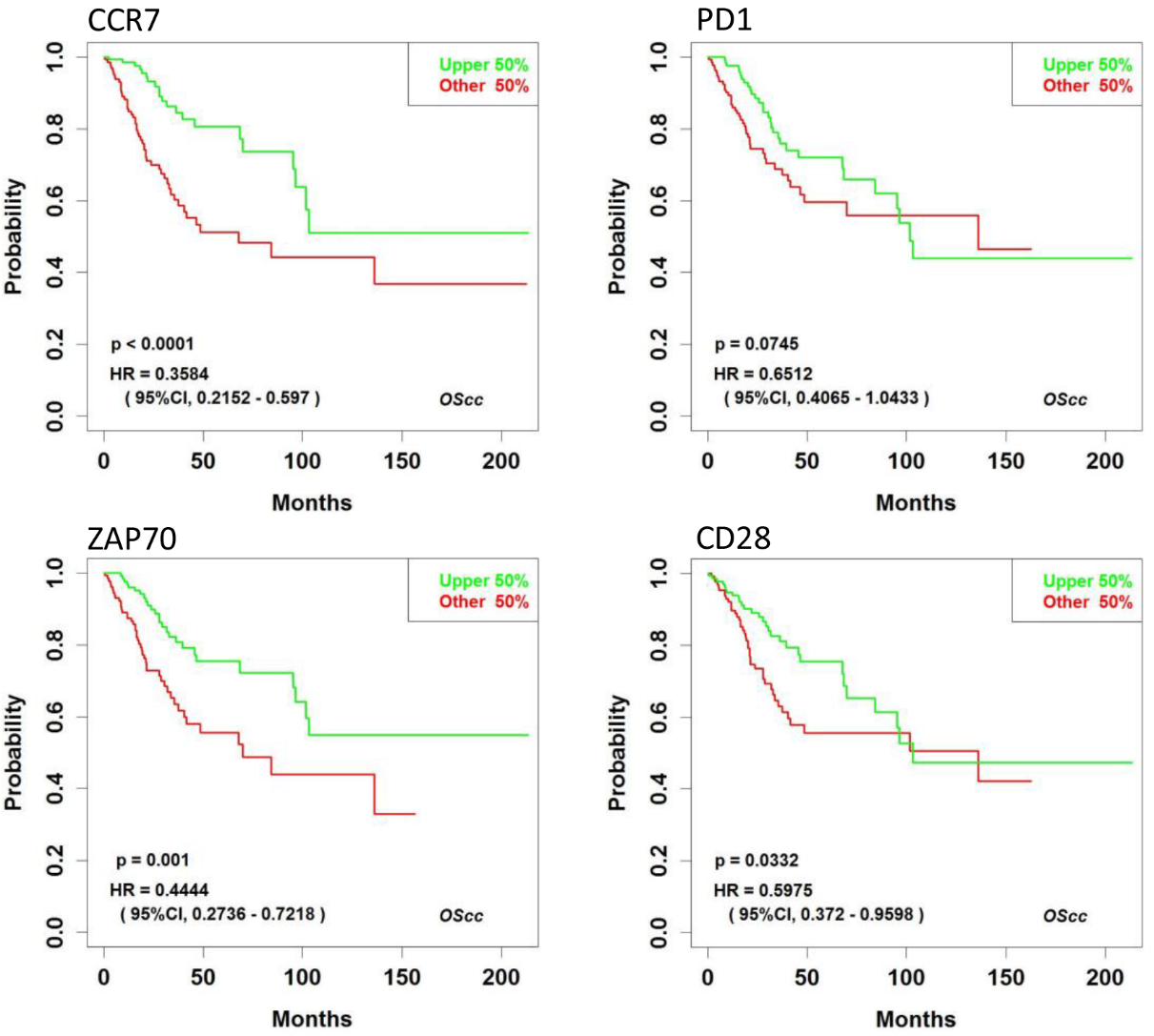
*

Supplementary figure 1. Kaplan-Meier survival curves and hazard ratios of CCR7, PD1, ZAP70, and CD28 in TCGA from OScc.
