# Supplementary material for: Integrative Bioinformatics Approaches to Screen Potential Prognostic Immune-Related Genes and Drugs in the Cervical Cancer Microenvironment
Source: Front Genet. 2020 Jul 7;11:727. doi: 10.3389/fgene.2020.00727 (PMC7359727; doi:10.3389/fgene.2020.00727)
Supplement: Supplementary file 2 [file Table_1.docx]

Supplementary Table.1 The clinicopathologic factors of cervical cancer patients in TCGA and GEO database

|  |  | TCGA  (N = 304) | GEO  (N = 55) |
| --- | --- | --- | --- |
| Factors | Group | Number (%) | Number (%) |
| Age | ≤ 45 years | 154 (50.7%) | 22 (40%) |
|  | > 45 years | 150 (49.3%) | 33 (60%) |
| FIGO stage | Stage ≦ II | 231 (76.0%) | 35 (63.6%) |
|  | Stage > II | 66 (21.7%) | 20 (36.4%) |
|  | Unknown | 7 (2.3%) |  |
| LNM | N0 | 133 (43.8%) |  |
|  | N1 | 60 (19.7%) |  |
|  | Unknown | 111 (36.5%) |  |
| HPV status | Negative | 169 (55.6%) |  |
|  | Positive | 9 (3.0%) |  |
|  | Unknown | 126 (41.4%) |  |
| Pathology | SCC | 253 (83.2%) | 51 (92.7%) |
|  | CAC | 47(15.5%) | 3 (5.5%) |
|  | ASC | 4 (1.3%) | 1 (1.8%) |

Abbreviations: LNM = lymphatic node metastasis, FIGO = International Federation of Gynecology and Obstetrics, SCC = cervical squamous carcinoma, CAC = cervical adenocarcinoma, ASC = cervical adenosquamous carcinoma, N0 = no lymphatic node metastasis, N1 = have lymphatic node metastasis.
