# Supplementary material for: Integrative Bioinformatics Approaches to Screen Potential Prognostic Immune-Related Genes and Drugs in the Cervical Cancer Microenvironment
Source: Front Genet. 2020 Jul 7;11:727. doi: 10.3389/fgene.2020.00727 (PMC7359727; doi:10.3389/fgene.2020.00727)
Supplement: Supplementary file 3 [file Table_2.docx]

Supplementary table 2. A total of 401 prognostic DEGS of cervical cancer and their expression level in TCGA

| Gene | Log2(FC) | Adjusted *p-*value | Expression level |
| --- | --- | --- | --- |
| *ABCB1* | 1.060 | < 0.001 | Up regulation |
| *ABCD2* | 1.832 | < 0.001 | Up regulation |
| *ACAP1* | 1.506 | < 0.001 | Up regulation |
| *ACKR1* | 1.299 | < 0.001 | Up regulation |
| *ACP5* | 1.402 | < 0.001 | Up regulation |
| *ACSM5* | 1.199 | < 0.001 | Up regulation |
| *ADA2* | 1.714 | < 0.001 | Up regulation |
| *ADGRF4* | 1.198 | < 0.001 | Up regulation |
| *ADRA1B* | -1.189 | < 0.001 | Down regulation |
| *AGAP2* | 1.173 | < 0.001 | Up regulation |
| *AIF1* | 1.973 | < 0.001 | Up regulation |
| *AIPL1* | 1.290 | < 0.001 | Up regulation |
| *AMPD1* | 1.177 | < 0.001 | Up regulation |
| *ANKRD55* | 1.315 | < 0.001 | Up regulation |
| *APBB1IP* | 1.896 | < 0.001 | Up regulation |
| *APOBEC3A* | 1.876 | < 0.001 | Up regulation |
| *APOBEC3D* | 1.100 | < 0.001 | Up regulation |
| *APOBEC3G* | 1.379 | < 0.001 | Up regulation |
| *APOBEC3H* | 1.459 | < 0.001 | Up regulation |
| APOBEC4 | -1.031 | 0.015 | Down regulation |
| *APOL3* | 1.913 | < 0.001 | Up regulation |
| *ARHGAP15* | 1.886 | < 0.001 | Up regulation |
| *ARHGAP25* | 1.273 | < 0.001 | Up regulation |
| *ARHGAP30* | 1.411 | < 0.001 | Up regulation |
| *ARHGAP9* | 1.854 | < 0.001 | Up regulation |
| *ARRDC5* | 1.115 | < 0.001 | Up regulation |
| ARSI | 1.021 | 0.001 | Up regulation |
| *ASGR2* | 1.410 | < 0.001 | Up regulation |
| *BATF* | 2.005 | < 0.001 | Up regulation |
| *BFSP2* | 1.475 | < 0.001 | Up regulation |
| *BIN2* | 1.500 | < 0.001 | Up regulation |
| *BLK* | 1.831 | < 0.001 | Up regulation |
| *BTK* | 1.750 | < 0.001 | Up regulation |
| *BTLA* | 2.021 | < 0.001 | Up regulation |
| *BTN3A3* | 1.117 | < 0.001 | Up regulation |
| C10orf82 | -1.244 | 0.007 | Down regulation |
| *C11orf21* | 1.942 | < 0.001 | Up regulation |
| *C16orf54* | 1.561 | < 0.001 | Up regulation |
| *C1orf194* | -1.901 | < 0.001 | Down regulation |

(Supplementary table 2. continue)

| *C3orf67* | -1.092 | < 0.001 | Down regulation |
| --- | --- | --- | --- |
| *C4orf50* | 1.065 | < 0.001 | Up regulation |
| *C5orf56* | 1.086 | < 0.001 | Up regulation |
| *C6orf118* | -1.865 | < 0.001 | Down regulation |
| *CACNA1D* | -1.291 | < 0.001 | Down regulation |
| *CALML3* | 2.407 | < 0.001 | Up regulation |
| *CARMIL2* | 1.234 | < 0.001 | Up regulation |
| *CCDC141* | 1.483 | < 0.001 | Up regulation |
| *CCDC69* | 1.037 | < 0.001 | Up regulation |
| *CCL19* | 2.576 | < 0.001 | Up regulation |
| *CCL22* | 1.293 | < 0.001 | Up regulation |
| *CCL23* | 1.182 | < 0.001 | Up regulation |
| *CCL25* | 1.096 | < 0.001 | Up regulation |
| *CCL5* | 2.425 | < 0.001 | Up regulation |
| *CCR2* | 2.120 | < 0.001 | Up regulation |
| *CCR5* | 2.260 | < 0.001 | Up regulation |
| *CCR7* | 1.784 | < 0.001 | Up regulation |
| *CD1A* | 1.785 | < 0.001 | Up regulation |
| *CD1B* | 1.740 | < 0.001 | Up regulation |
| *CD1C* | 1.459 | < 0.001 | Up regulation |
| *CD1E* | 1.603 | < 0.001 | Up regulation |
| *CD2* | 2.259 | < 0.001 | Up regulation |
| *CD200R1* | 1.745 | < 0.001 | Up regulation |
| *CD226* | 1.428 | < 0.001 | Up regulation |
| *CD247* | 1.876 | < 0.001 | Up regulation |
| *CD27* | 2.154 | < 0.001 | Up regulation |
| *CD28* | 1.734 | < 0.001 | Up regulation |
| *CD300LF* | 1.780 | < 0.001 | Up regulation |
| *CD37* | 1.761 | < 0.001 | Up regulation |
| *CD3D* | 2.414 | < 0.001 | Up regulation |
| *CD3E* | 2.207 | < 0.001 | Up regulation |
| *CD3G* | 2.228 | < 0.001 | Up regulation |
| *CD4* | 1.893 | < 0.001 | Up regulation |
| *CD40LG* | 1.713 | < 0.001 | Up regulation |
| *CD48* | 2.239 | < 0.001 | Up regulation |
| *CD5* | 1.915 | < 0.001 | Up regulation |
| *CD52* | 1.997 | < 0.001 | Up regulation |
| *CD53* | 1.884 | < 0.001 | Up regulation |
| *CD6* | 1.669 | < 0.001 | Up regulation |
| *CD7* | 2.195 | < 0.001 | Up regulation |
| *CD72* | 1.549 | < 0.001 | Up regulation |
| *CD79A* | 2.123 | < 0.001 | Up regulation |

(Supplementary table 2. continue)

| *CD79B* | 1.849 | < 0.001 | Up regulation |
| --- | --- | --- | --- |
| *CD80* | 1.785 | < 0.001 | Up regulation |
| *CD84* | 1.973 | < 0.001 | Up regulation |
| *CD86* | 1.862 | < 0.001 | Up regulation |
| *CD8A* | 2.381 | < 0.001 | Up regulation |
| *CD8B* | 1.755 | < 0.001 | Up regulation |
| *CD96* | 2.067 | < 0.001 | Up regulation |
| *CEACAM21* | 1.535 | < 0.001 | Up regulation |
| *CELF2* | 1.408 | < 0.001 | Up regulation |
| *CFP* | 1.039 | < 0.001 | Up regulation |
| *CHIT1* | 2.421 | < 0.001 | Up regulation |
| *CHST11* | 1.061 | < 0.001 | Up regulation |
| *CKB* | -1.098 | < 0.001 | Down regulation |
| *CLDN1* | 1.070 | < 0.001 | Up regulation |
| *CLEC10A* | 2.277 | < 0.001 | Up regulation |
| *CLEC12A* | 2.267 | < 0.001 | Up regulation |
| *CLEC4A* | 1.473 | < 0.001 | Up regulation |
| *CLEC6A* | 1.151 | < 0.001 | Up regulation |
| *CLEC9A* | 1.893 | < 0.001 | Up regulation |
| *CLECL1* | 1.949 | < 0.001 | Up regulation |
| *CLNK* | 1.850 | < 0.001 | Up regulation |
| *COCH* | -1.397 | < 0.001 | Down regulation |
| *COL6A5* | 1.106 | < 0.001 | Up regulation |
| *CORO1A* | 1.683 | < 0.001 | Up regulation |
| *CPVL* | 1.171 | < 0.001 | Up regulation |
| CSAG2 | 1.080 | 0.014 | Up regulation |
| CSAG3 | 1.026 | 0.019 | Up regulation |
| *CSF2RA* | 1.527 | < 0.001 | Up regulation |
| *CSF2RB* | 1.658 | < 0.001 | Up regulation |
| *CST7* | 2.170 | < 0.001 | Up regulation |
| *CTLA4* | 2.264 | < 0.001 | Up regulation |
| *CXCL10* | 2.940 | < 0.001 | Up regulation |
| *CXCL9* | 2.970 | < 0.001 | Up regulation |
| *CXCR3* | 2.270 | < 0.001 | Up regulation |
| *CXCR6* | 2.135 | < 0.001 | Up regulation |
| *CXorf21* | 1.637 | < 0.001 | Up regulation |
| *CXorf65* | 1.468 | < 0.001 | Up regulation |
| *CYBB* | 2.019 | < 0.001 | Up regulation |
| CYP4B1 | 1.007 | 0.021 | Up regulation |
| *CYSLTR1* | 1.016 | < 0.001 | Up regulation |
| *CYSLTR2* | 1.423 | < 0.001 | Up regulation |
| *CYTH4* | 1.683 | < 0.001 | Up regulation |

(Supplementary table 2. continue)

| *CYTIP* | 1.885 | < 0.001 | Up regulation |
| --- | --- | --- | --- |
| *DMRT2* | -1.505 | < 0.001 | Down regulation |
| *DNAJC5B* | 1.577 | < 0.001 | Up regulation |
| *DNASE1L3* | 1.318 | < 0.001 | Up regulation |
| *DOC2B* | 1.031 | < 0.001 | Up regulation |
| *DOCK2* | 1.848 | < 0.001 | Up regulation |
| *DOK2* | 1.959 | < 0.001 | Up regulation |
| *DPEP2* | 1.249 | < 0.001 | Up regulation |
| *DTHD1* | 1.091 | < 0.001 | Up regulation |
| *ELAVL4* | 1.390 | < 0.001 | Up regulation |
| *ELMO1* | 1.182 | < 0.001 | Up regulation |
| *EOMES* | 2.011 | < 0.001 | Up regulation |
| ERICH3 | -1.054 | 0.016 | Down regulation |
| *ETV7* | 1.389 | < 0.001 | Up regulation |
| *EVI2A* | 1.792 | < 0.001 | Up regulation |
| *EVI2B* | 1.862 | < 0.001 | Up regulation |
| *FAM49A* | 1.073 | < 0.001 | Up regulation |
| *FATE1* | 1.021 | < 0.001 | Up regulation |
| FCER1A | 1.086 | 0.001 | Up regulation |
| *FCER1G* | 1.906 | < 0.001 | Up regulation |
| *FCER2* | 1.149 | < 0.001 | Up regulation |
| *FCGR1A* | 2.203 | < 0.001 | Up regulation |
| *FCGR2B* | 1.774 | < 0.001 | Up regulation |
| *FCMR* | 1.045 | < 0.001 | Up regulation |
| *FCRL1* | 1.319 | < 0.001 | Up regulation |
| *FCRL2* | 1.088 | < 0.001 | Up regulation |
| *FCRL4* | 1.241 | < 0.001 | Up regulation |
| *FCRL5* | 2.096 | < 0.001 | Up regulation |
| *FCRL6* | 1.873 | < 0.001 | Up regulation |
| *FERMT3* | 1.687 | < 0.001 | Up regulation |
| *FGD2* | 1.192 | < 0.001 | Up regulation |
| *FGD3* | 1.061 | < 0.001 | Up regulation |
| *FGR* | 1.197 | < 0.001 | Up regulation |
| *FLT3* | 1.778 | < 0.001 | Up regulation |
| *FLT3LG* | 1.015 | < 0.001 | Up regulation |
| FOXJ1 | -1.140 | 0.026 | Down regulation |
| *FOXN1* | 1.271 | < 0.001 | Up regulation |
| *FOXP3* | 1.443 | < 0.001 | Up regulation |
| *FUT7* | 2.231 | < 0.001 | Up regulation |
| *FZD8* | -1.381 | < 0.001 | Down regulation |
| *GAB3* | 1.564 | < 0.001 | Up regulation |
| *GBP1* | 1.959 | < 0.001 | Up regulation |

(Supplementary table 2. continue)

| *GBP4* | 2.276 | < 0.001 | Up regulation |
| --- | --- | --- | --- |
| *GBP5* | 2.664 | < 0.001 | Up regulation |
| *GIMAP1* | 1.672 | < 0.001 | Up regulation |
| *GIMAP4* | 1.718 | < 0.001 | Up regulation |
| *GIMAP7* | 1.670 | < 0.001 | Up regulation |
| *GLYATL2* | -1.724 | < 0.001 | Down regulation |
| *GMFG* | 1.717 | < 0.001 | Up regulation |
| *GNG2* | 1.123 | < 0.001 | Up regulation |
| *GNG8* | 1.160 | < 0.001 | Up regulation |
| *GNGT2* | 1.819 | < 0.001 | Up regulation |
| *GPR171* | 2.117 | < 0.001 | Up regulation |
| *GPR18* | 1.925 | < 0.001 | Up regulation |
| *GPR183* | 1.622 | < 0.001 | Up regulation |
| *GPR82* | 1.656 | < 0.001 | Up regulation |
| *GRAP2* | 1.891 | < 0.001 | Up regulation |
| *GTSF1* | 1.399 | < 0.001 | Up regulation |
| *GYPC* | 1.439 | < 0.001 | Up regulation |
| *GZMH* | 2.720 | < 0.001 | Up regulation |
| *GZMK* | 2.656 | < 0.001 | Up regulation |
| *GZMM* | 2.312 | < 0.001 | Up regulation |
| *HAMP* | 1.668 | < 0.001 | Up regulation |
| *HAPLN3* | 1.028 | < 0.001 | Up regulation |
| *HAVCR2* | 1.977 | < 0.001 | Up regulation |
| *HCK* | 1.449 | < 0.001 | Up regulation |
| *HCLS1* | 1.194 | < 0.001 | Up regulation |
| *HEATR9* | 1.420 | < 0.001 | Up regulation |
| *HLA-DMB* | 1.504 | < 0.001 | Up regulation |
| *HLA-DOB* | 1.334 | < 0.001 | Up regulation |
| *HLA-DPB1* | 1.950 | < 0.001 | Up regulation |
| *HLA-DQA1* | 2.253 | < 0.001 | Up regulation |
| *HLA-DQA2* | 2.060 | < 0.001 | Up regulation |
| *HLA-DQB1* | 1.819 | < 0.001 | Up regulation |
| *HLA-DRA* | 1.874 | < 0.001 | Up regulation |
| HTR3A | -1.219 | 0.002 | Down regulation |
| *HTRA4* | 1.551 | < 0.001 | Up regulation |
| *ICOS* | 2.229 | < 0.001 | Up regulation |
| *IDO2* | 1.399 | < 0.001 | Up regulation |
| *IFI30* | 1.111 | < 0.001 | Up regulation |
| *IGLL5* | 2.408 | < 0.001 | Up regulation |
| *IGSF6* | 1.728 | < 0.001 | Up regulation |
| *IKZF1* | 1.864 | < 0.001 | Up regulation |
| *IKZF3* | 1.219 | < 0.001 | Up regulation |

(Supplementary table 2. continue)

| *IL10RA* | 1.844 | < 0.001 | Up regulation |
| --- | --- | --- | --- |
| *IL12B* | 1.413 | < 0.001 | Up regulation |
| *IL12RB1* | 2.119 | < 0.001 | Up regulation |
| *IL16* | 1.668 | < 0.001 | Up regulation |
| *IL18BP* | 1.084 | < 0.001 | Up regulation |
| *IL18RAP* | 1.817 | < 0.001 | Up regulation |
| *IL21R* | 2.317 | < 0.001 | Up regulation |
| *IL22RA2* | 1.699 | < 0.001 | Up regulation |
| *IL2RA* | 1.927 | < 0.001 | Up regulation |
| *IL2RG* | 2.109 | < 0.001 | Up regulation |
| *INPP5D* | 1.039 | < 0.001 | Up regulation |
| *IRF1* | 1.344 | < 0.001 | Up regulation |
| *IRF4* | 1.880 | < 0.001 | Up regulation |
| *ITGAL* | 2.131 | < 0.001 | Up regulation |
| *ITK* | 1.913 | < 0.001 | Up regulation |
| *JAK3* | 1.435 | < 0.001 | Up regulation |
| *JAKMIP1* | 2.363 | < 0.001 | Up regulation |
| *JAKMIP2* | 1.110 | < 0.001 | Up regulation |
| *JAML* | 2.043 | < 0.001 | Up regulation |
| *JCHAIN* | 2.153 | < 0.001 | Up regulation |
| *KCNA3* | 1.655 | < 0.001 | Up regulation |
| *KCNJ10* | 1.377 | < 0.001 | Up regulation |
| *KLHL6* | 1.519 | < 0.001 | Up regulation |
| *KLRB1* | 1.606 | < 0.001 | Up regulation |
| *KLRC2* | 1.550 | < 0.001 | Up regulation |
| *KLRD1* | 2.258 | < 0.001 | Up regulation |
| *KLRK1* | 1.405 | < 0.001 | Up regulation |
| *LAPTM5* | 1.833 | < 0.001 | Up regulation |
| *LAT2* | 1.252 | < 0.001 | Up regulation |
| *LAX1* | 1.733 | < 0.001 | Up regulation |
| *LCK* | 1.520 | < 0.001 | Up regulation |
| *LGALS2* | 1.585 | < 0.001 | Up regulation |
| *LILRA4* | 2.084 | < 0.001 | Up regulation |
| *LILRB1* | 1.894 | < 0.001 | Up regulation |
| *LILRB4* | 2.195 | < 0.001 | Up regulation |
| *LRMP* | 1.284 | < 0.001 | Up regulation |
| *LSP1* | 1.739 | < 0.001 | Up regulation |
| *LST1* | 1.878 | < 0.001 | Up regulation |
| *LTB* | 1.935 | < 0.001 | Up regulation |
| *LY9* | 2.255 | < 0.001 | Up regulation |
| *LY96* | 1.558 | < 0.001 | Up regulation |
| *MAP4K1* | 1.530 | < 0.001 | Up regulation |

(Supplementary table 2. continue)

| *MFNG* | 1.090 | < 0.001 | Up regulation |
| --- | --- | --- | --- |
| *MMP25* | 1.306 | < 0.001 | Up regulation |
| *MPEG1* | 1.886 | < 0.001 | Up regulation |
| *MS4A4A* | 1.776 | < 0.001 | Up regulation |
| *MS4A4E* | 1.244 | < 0.001 | Up regulation |
| *MS4A6A* | 1.946 | < 0.001 | Up regulation |
| MUC21 | 1.150 | 0.023 | Up regulation |
| *MYO1F* | 1.688 | < 0.001 | Up regulation |
| *MZB1* | 2.147 | < 0.001 | Up regulation |
| *NCF1* | 1.668 | < 0.001 | Up regulation |
| *NCF4* | 1.267 | < 0.001 | Up regulation |
| *NCKAP1L* | 1.939 | < 0.001 | Up regulation |
| *NCR1* | 1.843 | < 0.001 | Up regulation |
| NEXMIF | -1.110 | 0.017 | Down regulation |
| *NFAM1* | 1.444 | < 0.001 | Up regulation |
| *NIBAN3* | 1.194 | < 0.001 | Up regulation |
| *NLRC3* | 1.310 | < 0.001 | Up regulation |
| *NLRC5* | 1.062 | < 0.001 | Up regulation |
| *NUGGC* | 1.710 | < 0.001 | Up regulation |
| *OASL* | 1.290 | < 0.001 | Up regulation |
| *OTOA* | 1.114 | < 0.001 | Up regulation |
| *P2RX1* | 1.178 | < 0.001 | Up regulation |
| *P2RY10* | 1.964 | < 0.001 | Up regulation |
| *P2RY13* | 1.977 | < 0.001 | Up regulation |
| *P2RY8* | 1.706 | < 0.001 | Up regulation |
| *PARP15* | 1.492 | < 0.001 | Up regulation |
| *PDCD1* | 2.245 | < 0.001 | Up regulation |
| *PIK3R5* | 1.509 | < 0.001 | Up regulation |
| *PILRA* | 1.261 | < 0.001 | Up regulation |
| *PKD2L1* | 1.281 | < 0.001 | Up regulation |
| *PLA2G2D* | 3.259 | < 0.001 | Up regulation |
| *PLA2G7* | 1.624 | < 0.001 | Up regulation |
| *PLAAT4* | 1.439 | < 0.001 | Up regulation |
| *PLAC8* | 1.214 | < 0.001 | Up regulation |
| *PLCB2* | 1.168 | < 0.001 | Up regulation |
| *PLD4* | 1.738 | < 0.001 | Up regulation |
| *PNOC* | 1.484 | < 0.001 | Up regulation |
| *POU2AF1* | 1.670 | < 0.001 | Up regulation |
| *PPP1R16B* | 1.451 | < 0.001 | Up regulation |
| *PRAM1* | 1.345 | < 0.001 | Up regulation |
| *PRKAA2* | -1.715 | < 0.001 | Down regulation |
| *PRKCB* | 1.768 | < 0.001 | Up regulation |

(Supplementary table 2. continue)

| *PSMB10* | 1.131 | < 0.001 | Up regulation |
| --- | --- | --- | --- |
| *PSTPIP1* | 1.882 | < 0.001 | Up regulation |
| *PTCRA* | 1.694 | < 0.001 | Up regulation |
| *PTGDR* | 1.074 | < 0.001 | Up regulation |
| *PTGDS* | 1.470 | < 0.001 | Up regulation |
| *PTPN22* | 1.508 | < 0.001 | Up regulation |
| *PTPN7* | 1.972 | < 0.001 | Up regulation |
| *PYHIN1* | 2.151 | < 0.001 | Up regulation |
| *RAB33A* | 1.480 | < 0.001 | Up regulation |
| *RAB39B* | 1.214 | < 0.001 | Up regulation |
| *RAB3B* | -1.234 | < 0.001 | Down regulation |
| *RASAL3* | 1.873 | < 0.001 | Up regulation |
| *RASGRP1* | 1.358 | < 0.001 | Up regulation |
| *RASGRP2* | 1.165 | < 0.001 | Up regulation |
| *RASSF4* | 1.324 | < 0.001 | Up regulation |
| *RCSD1* | 1.486 | < 0.001 | Up regulation |
| *RENBP* | 1.267 | < 0.001 | Up regulation |
| *RGS1* | 1.733 | < 0.001 | Up regulation |
| *RGS18* | 1.705 | < 0.001 | Up regulation |
| *RHOH* | 1.642 | < 0.001 | Up regulation |
| *RIPOR2* | 1.267 | < 0.001 | Up regulation |
| *ROPN1B* | -1.169 | < 0.001 | Down regulation |
| *RTN1* | 1.056 | < 0.001 | Up regulation |
| *RTP4* | 1.339 | < 0.001 | Up regulation |
| *RUFY4* | 2.049 | < 0.001 | Up regulation |
| *S100B* | 1.877 | < 0.001 | Up regulation |
| *S1PR4* | 1.517 | < 0.001 | Up regulation |
| *SAMSN1* | 1.699 | < 0.001 | Up regulation |
| *SASH3* | 1.963 | < 0.001 | Up regulation |
| *SBK1* | -1.246 | < 0.001 | Down regulation |
| *SCML4* | 2.191 | < 0.001 | Up regulation |
| *SCUBE1* | 1.442 | < 0.001 | Up regulation |
| *SDS* | 1.210 | < 0.001 | Up regulation |
| *SELL* | 1.716 | < 0.001 | Up regulation |
| *SELP* | 1.125 | < 0.001 | Up regulation |
| *SELPLG* | 1.579 | < 0.001 | Up regulation |
| *SEPTIN1* | 1.459 | < 0.001 | Up regulation |
| *SH2D1A* | 2.400 | < 0.001 | Up regulation |
| *SHANK2* | -1.254 | < 0.001 | Down regulation |
| *SIGLEC1* | 2.189 | < 0.001 | Up regulation |
| *SIGLEC10* | 1.882 | < 0.001 | Up regulation |
| *SIRPG* | 2.477 | < 0.001 | Up regulation |

(Supplementary table 2. continue)

| *SIT1* | 2.208 | < 0.001 | Up regulation |
| --- | --- | --- | --- |
| *SLA* | 1.857 | < 0.001 | Up regulation |
| *SLA2* | 2.195 | < 0.001 | Up regulation |
| *SLAMF1* | 2.034 | < 0.001 | Up regulation |
| *SLAMF6* | 2.215 | < 0.001 | Up regulation |
| *SLAMF7* | 2.140 | < 0.001 | Up regulation |
| *SLAMF8* | 2.013 | < 0.001 | Up regulation |
| *SLC12A3* | 1.536 | < 0.001 | Up regulation |
| SLC13A2 | -1.017 | 0.010 | Down regulation |
| *SLC15A3* | 1.090 | < 0.001 | Up regulation |
| *SLFN12L* | 1.358 | < 0.001 | Up regulation |
| *SNAI3* | 1.025 | < 0.001 | Up regulation |
| *SNX20* | 2.111 | < 0.001 | Up regulation |
| *SP140* | 2.079 | < 0.001 | Up regulation |
| *SPI1* | 1.833 | < 0.001 | Up regulation |
| *SPIB* | 1.931 | < 0.001 | Up regulation |
| *SPN* | 1.869 | < 0.001 | Up regulation |
| *SPOCK2* | 1.501 | < 0.001 | Up regulation |
| SPTSSB | -1.081 | 0.001 | Down regulation |
| *SSTR3* | 1.698 | < 0.001 | Up regulation |
| *ST8SIA4* | 1.191 | < 0.001 | Up regulation |
| *STAP1* | 1.560 | < 0.001 | Up regulation |
| SYT13 | -1.850 | 0.001 | Down regulation |
| *SYT6* | 1.159 | < 0.001 | Up regulation |
| *TBC1D10C* | 1.990 | < 0.001 | Up regulation |
| *TBX21* | 2.189 | < 0.001 | Up regulation |
| *TCL1A* | 1.948 | < 0.001 | Up regulation |
| *TESPA1* | 1.920 | < 0.001 | Up regulation |
| *TFEC* | 1.926 | < 0.001 | Up regulation |
| *THEMIS* | 1.838 | < 0.001 | Up regulation |
| *TIFAB* | 2.378 | < 0.001 | Up regulation |
| *TIGIT* | 2.369 | < 0.001 | Up regulation |
| *TIMD4* | 1.555 | < 0.001 | Up regulation |
| *TLR10* | 1.687 | < 0.001 | Up regulation |
| *TMC8* | 1.220 | < 0.001 | Up regulation |
| *TMEM273* | 1.259 | < 0.001 | Up regulation |
| TMEM82 | -1.099 | 0.003 | Down regulation |
| *TNFAIP8L2* | 1.963 | < 0.001 | Up regulation |
| *TNFRSF13B* | 2.199 | < 0.001 | Up regulation |
| *TNFRSF1B* | 1.458 | < 0.001 | Up regulation |
| *TNFRSF8* | 1.502 | < 0.001 | Up regulation |
| *TNFSF13B* | 1.839 | < 0.001 | Up regulation |

(Supplementary table 2. continue)

| *TNFSF8* | 1.560 | < 0.001 | Up regulation |
| --- | --- | --- | --- |
| *TPSB2* | 1.191 | < 0.001 | Up regulation |
| *TRAT1* | 2.217 | < 0.001 | Up regulation |
| *TREM2* | 1.887 | < 0.001 | Up regulation |
| *TREML1* | 1.525 | < 0.001 | Up regulation |
| *TRIM22* | 1.462 | < 0.001 | Up regulation |
| TRIM54 | -1.540 | 0.001 | Down regulation |
| *TSHR* | 1.062 | < 0.001 | Up regulation |
| *TSPAN32* | 1.541 | < 0.001 | Up regulation |
| *TTC24* | 1.910 | < 0.001 | Up regulation |
| *TYROBP* | 1.951 | < 0.001 | Up regulation |
| *UBA7* | 1.054 | < 0.001 | Up regulation |
| *UBASH3A* | 2.039 | < 0.001 | Up regulation |
| *UBD* | 2.464 | < 0.001 | Up regulation |
| *UBE2L6* | 1.119 | < 0.001 | Up regulation |
| *UMODL1* | -1.126 | < 0.001 | Down regulation |
| *VCAM1* | 1.346 | < 0.001 | Up regulation |
| VTCN1 | -1.228 | 0.003 | Down regulation |
| *WAS* | 1.765 | < 0.001 | Up regulation |
| *XCR1* | 1.674 | < 0.001 | Up regulation |
| *ZAP70* | 1.916 | < 0.001 | Up regulation |
| ZBBX | -1.583 | 0.002 | Down regulation |
| *ZBTB32* | 1.198 | < 0.001 | Up regulation |
| *ZC3H12D* | 1.055 | < 0.001 | Up regulation |
| *ZNF683* | 2.740 | < 0.001 | Up regulation |
| *ZNF831* | 2.143 | < 0.001 | Up regulation |
