# Supplementary material for: Integrative Bioinformatics Approaches to Screen Potential Prognostic Immune-Related Genes and Drugs in the Cervical Cancer Microenvironment
Source: Front Genet. 2020 Jul 7;11:727. doi: 10.3389/fgene.2020.00727 (PMC7359727; doi:10.3389/fgene.2020.00727)
Supplement: Supplementary file 4 [file Table_3.docx]

Supplementary Table 3. Seventy-nine key prognostic DEGs extracted from PPI network

| Gene | *p*-value |
| --- | --- |
| *APBB1IP* | 0.017 |
| *BLK* | 0.009 |
| *BTK* | 0.014 |
| *CCL19* | 0.039 |
| *CCL25* | 0.020 |
| *CCL5* | 0.013 |
| *CCR2* | < 0.001 |
| *CCR5* | 0.039 |
| *CCR7* | < 0.001 |
| *CD247* | 0.011 |
| *CD28* | 0.006 |
| *CD3D* | < 0.001 |
| *CD3E* | 0.005 |
| *CD3G* | < 0.001 |
| *CD4* | 0.047 |
| *CD40LG* | 0.006 |
| *CD53* | 0.041 |
| *CD79B* | 0.001 |
| *CD80* | 0.010 |
| *CD86* | 0.021 |
| *CD8A* | 0.008 |
| *CD8B* | 0.013 |
| *CLEC12A* | 0.045 |
| *CTLA4* | 0.006 |
| *CXCL10* | 0.038 |
| *CXCL9* | 0.005 |
| *CXCR3* | 0.002 |
| *CXCR6* | 0.002 |
| *CYBB* | 0.003 |
| *FCER1G* | 0.026 |
| *FCER2* | 0.009 |
| *FCGR1A* | 0.018 |
| *FCGR2B* | 0.010 |
| *FGR* | 0.037 |
| *FOXP3* | < 0.001 |
| *GBP1* | 0.040 |
| *GBP4* | 0.045 |
| *GBP5* | 0.044 |
| *GNG2* | 0.023 |

(Supplementary Table 3. continued)

| *GNG8* | 0.003 |
| --- | --- |
| *GNGT2* | 0.035 |
| *GPR18* | 0.017 |
| *GPR183* | 0.016 |
| *GRAP2* | < 0.001 |
| *HCK* | 0.019 |
| *HLA-DPB1* | 0.012 |
| *HLA-DQA1* | 0.003 |
| *HLA-DQA2* | 0.015 |
| *HLA-DQB1* | 0.040 |
| *HLA-DRA* | 0.029 |
| *IFI30* | 0.006 |
| *IGLL5* | 0.008 |
| *IL2RA* | 0.009 |
| *IL2RG* | 0.030 |
| *INPP5D* | 0.003 |
| *IRF1* | 0.018 |
| *IRF4* | 0.004 |
| *ITGAL* | 0.028 |
| *ITK* | 0.007 |
| *JAK3* | < 0.001 |
| *LCK* | 0.001 |
| *MMP25* | 0.041 |
| *OASL* | 0.048 |
| *P2RX1* | 0.022 |
| *P2RY13* | 0.002 |
| *PD1* | 0.032 |
| *PNOC* | 0.003 |
| *PRKCB* | 0.001 |
| *PTPN22* | 0.019 |
| *RHOH* | 0.003 |
| *S1PR4* | < 0.001 |
| *SPI1* | 0.016 |
| *SSTR3* | 0.044 |
| *TNFRSF1B* | 0.035 |
| *TRIM22* | 0.033 |
| *TYROBP* | 0.008 |
| *VCAM1* | 0.002 |
| *WAS* | 0.002 |
| *ZAP70* | 0.001 |
