# Supplementary material for: Integrative Bioinformatics Approaches to Screen Potential Prognostic Immune-Related Genes and Drugs in the Cervical Cancer Microenvironment
Source: Front Genet. 2020 Jul 7;11:727. doi: 10.3389/fgene.2020.00727 (PMC7359727; doi:10.3389/fgene.2020.00727)
Supplement: Supplementary file 5 [file Table_4.docx]

|  | | TCGA | | |  | GEO | | |
| --- | --- | --- | --- | --- | --- | --- | --- | --- |
| Covariate | | HR | 95% CI | *P*-value |  | HR | 95% CI | *P*-value |
| Age | ≤ 45 years | Reference | | |  | Reference | | |
|  | > 45 years | 1.21 | 0.73-1.99 | 0.465 |  | 1.12 | 0.46-2.70 | 0.801 |
| LNM | N0 | Reference | | |  | Reference | | |
|  | N1 | 2.37 | 1.12-5.05 | 0.025 |  |  |  |  |
|  | UNKNOWN | 3.46 | 1.87-6.42 | **< 0.001** |  |  |  |  |
| FIGO | ≤ Ⅱ | Reference | | |  | Reference | | |
|  | > Ⅱ | 2.44 | 1.44-4.09 | **< 0.001** |  | 6.16 | 2.47-15.39 | **< 0.001** |
|  | UNKNOWN | 0.00 | 0.00-Infinitive | 0.992 |  |  |  |  |
| pathology | SCC | Reference | | |  | Reference |  |  |
|  | CAC/ ASC | 0.95 | 0.48-1.87 | 0.89 |  | 0.49 | 0.07-3.72 | 0.497 |
| *CCR7* | Low | Reference | | |  | Reference | | |
|  | High | 0.31 | 0.17-0.53 | **< 0.001** |  | 0.31 | 0.12-0.79 | **0.015** |
| *PD1* | Low | Reference | | |  | Reference | | |
|  | High | 0.58 | 0.35-0.9594 | **0.033** |  | 0.316 | 0.12-0.82 | **0.017** |
| *ZAP70* | Low | Reference | | |  | Reference |  |  |
|  | High | 0.41 | 0.25-0.69 | **< 0.001** |  | 0.328 | 0.13-0.84 | **0.021** |
| *CD28* | Low | Reference | | |  | Reference | | |
|  | High | 0.49 | 0.29-0.83 | **0.007** |  | 0.41 | 0.16-1.02 | 0.055 |

Supplementary Table 4. Univariate cox regression analysis for clinicopathological factors and four validated genes (*CCR7, PD1, ZAP70*, and *CD28*) in TCGA and GEO.

Abbreviations: LNM = lymphatic node metastasis, FIGO = International Federation of Gynecology and Obstetrics stage, SCC = cervical squamous carcinoma, CAC = cervical adenocarcinoma, ASC = cervical adenosquamous carcinoma, HR = Hazard Ratio, CI = Confidence Interval, N0 = no lymphatic node metastasis, N1 = have lymphatic node metastasis.
